# Supplementary material for: A new transgenic reporter line reveals Wnt-dependent Snai2 re-expression and cranial neural crest differentiation in Xenopus
Source: Sci Rep. 2019 Aug 1;9:11191. doi: 10.1038/s41598-019-47665-9 (PMC6672020; doi:10.1038/s41598-019-47665-9)
Supplement: Supplementary file 1 — Supplementary information [file 41598_2019_47665_MOESM1_ESM.docx]

**Supplemental information**

**A new transgenic reporter line reveals Wnt-dependent Snai2 re-expression and cranial neural crest differentiation in *Xenopus***

Jiejing Li, Mark Perfetto, Christopher Materna, Rebecca Li, Hong Thi Tran, Kris Vleminckx, Melinda K. Duncan and Shuo Wei


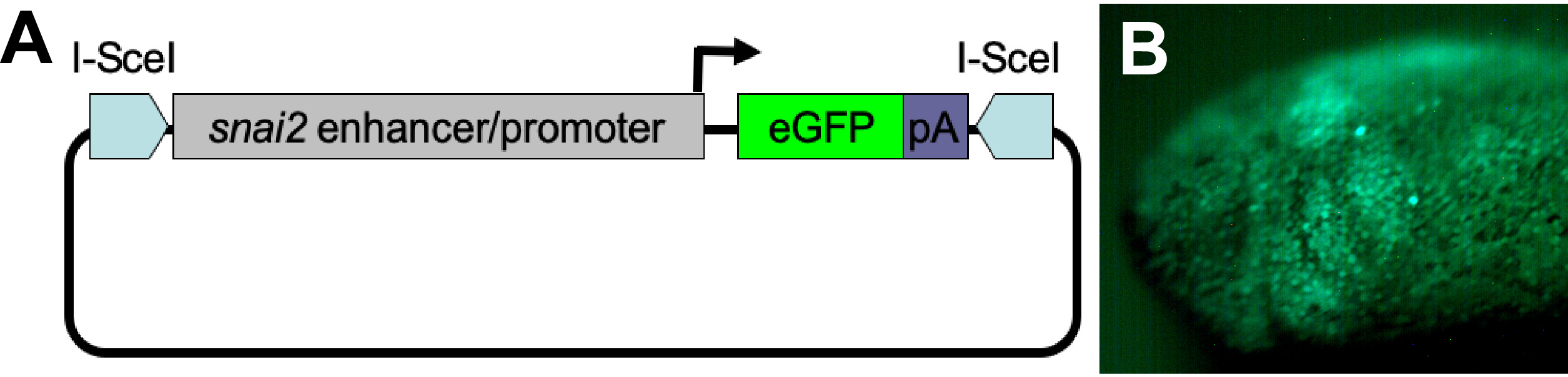


**Supplemental Fig. S1. Generation of the *snai2:eGFP* transgenic line. A.** The transgenic construct that was used for generating the *snai2:eGFP* line. pA, SV40 polyadenylation site. **B.** A *snai2:eGFP* transgenic founder showing eGFP expression in the migrating CNC streams.


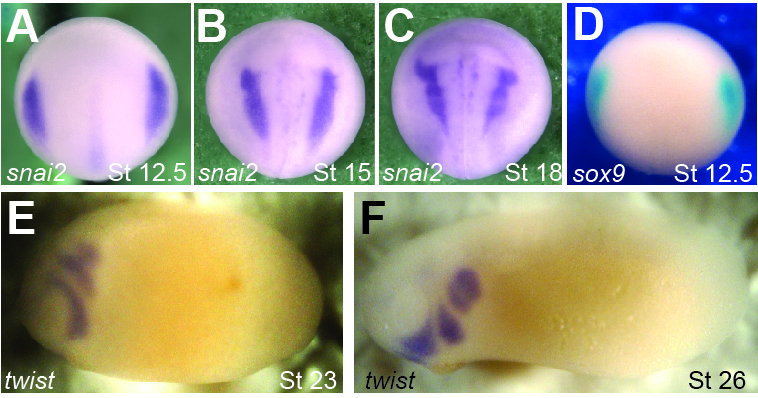


**Supplemental Fig. S2. Normal marker expression in the pre-migratory and migrating CNC in heterozygous *snai2:eGFP* transgenic embryos.** Heterozygous *snai2:eGFP* embryos were fixed at the indicated stages, and in situ hybridization was carried out for *snai2* (**A**-**C**), *sox9* (**D**) or *twist* (**E** and **F**). Embryos are shown in dorsal view with anterior at the top (**A**-**D**) or side view with anterior to the left (**E** and **F**).


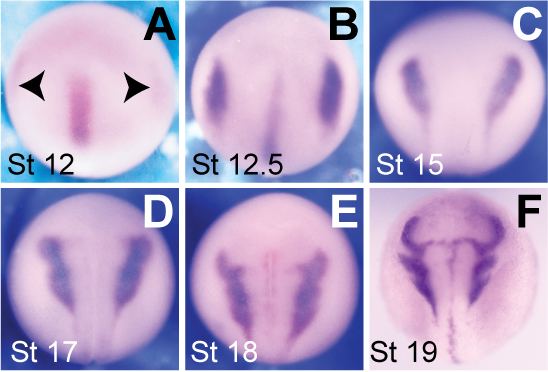


**Supplemental Fig. S3. The transcripts of *snai2* are expressed in pre-migratory and migrating CNC.** In situ hybridization was performed for *snai2* with wild-type *X. tropicalis* embryos at the indicated stages. Arrowheads in **A** indicate weak *snai2* expression in the emerging CNC. *Snai2* continues to be expressed in the pre-migratory (**B** and **C**) as well as migrating (**D**-**F**) CNC cells. Embryos are shown in dorsal view with anterior at the top.


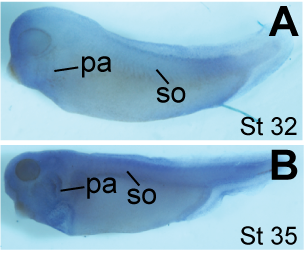


**Supplemental Fig. S4. Expression of *egfp* mRNA during early CNC differentiation.** In situ hybridization was carried out for *eGFP* with *snai2:eGFP* embryos at the indicated stages. Note the *eGFP* expression in the pharyngeal arches (pa) and somites (so). Embryos are shown in side view with anterior to the left.

**Table S1 Primers used for cloning and subcloning of the *snai2* promoter/enhancer**

| Primer name | Sequence (5’-3’) |
| --- | --- |
| *Snai2* forward | GGCCCTGTACATTGTTGG |
| *Snai2* reverse | AGAGGCACAGGATCTGCATTACTG |
| IS-*snai2*:eGFP SalI forward | ATATGTCGACGGCCCTGTACATTGTTGG |
| IS-*snai2*:eGFP NotI reverse | ATATGCGGCCGCTCACCAAGTGGCAGCGCT |

Restriction sites that were used to subclone the *snai2* promoter/enhancer into the IS-eGFP vector are underscored.
